# Noninvasive Assessment of Interstitial Fibrosis and Tubular Atrophy in Renal Transplant by Combining Point-Shear Wave Elastography and Estimated Glomerular Filtration Rate

**DOI:** 10.3390/diagnostics12010018

**Published:** 2021-12-23

**Authors:** Chi Qin, Hailong Jin, Haixiang Zhang, Yun Zhang, Zhaojie Guan, Yongyan Gao

**Affiliations:** 1 The Training Site for Postgraduate of Jinzhou Medical University, Department of Ultrasound, The Third Medical Center of Chinese PLA General Hospital, 69 Yongding Road, Hai Dian, Beijing 100039, China; 13115219226@163.com; 2 Department of Organ Transplantation, The Third Medical Center of Chinese PLA General Hospital, 69 Yongding Road, Hai Dian, Beijing 100039, China; guanzhaojie@126.com; 3 Department of Ultrasound, The Third Medical Center of Chinese PLA General Hospital, 69 Yongding Road, Hai Dian, Beijing 100039, China; zhanghaixiang1217@163.com (H.Z.); zy826840491@163.com (Y.Z.)

**Keywords:** point-shear wave elastography, interstitial fibrosis and tubular atrophy, estimated glomerular filtration, renal transplantation

## Abstract

The purpose of this study was to evaluate the feasibility of the combination of point-shear wave elastography (p-SWE) and estimated glomerular filtration rate (eGFR) for assessing different stages of interstitial fibrosis and tubular atrophy (IF/TA) in patients with chronic renal allograft dysfunction (CAD). From September 2020 to August 2021, 47 patients who underwent renal biopsy and p-SWE examinations were consecutively enrolled in this study. The areas under the receiver operating characteristic curves (AUCs) were calculated to evaluate overall accuracy and to identify the optimal cutoff values for different IF/TA stages. A total of 43 patients were enrolled in this study. The renal cortical stiffness and eGFR showed a significant difference between IF/TA Grade 0–1 and Grade 2–3 (*p* < 0.001). Additionally, renal stiffness and eGFR were independent predictors for moderate-to-severe IF/TA (Grade ≥ 2) according to multiple logistic regression analysis. The combination of p-SWE and eGFR, with an optimal cutoff value of −1.63, was superior to eGFR alone in assessing moderate-to-severe interstitial fibrosis (AUC, 0.86 vs. 0.72, *p* = 0.02) or tubular atrophy (AUC, 0.88 vs. 0.74, *p* = 0.02). There was no difference between p-SWE and eGFR in assessing moderate-to-severe IF/TA (AUC, 0.85 vs. 0.79, *p* = 0.61). Therefore, combining p-SWE and eGFR is worthy of clinical popularization and application.

## 1. Introduction

Renal transplantation (RT) is an effective tool for the treatment of end-stage renal disease [1]. With the improvement in RT techniques and application of immunosuppressors, the 1-year survival rate for patients with RT has reached 95% [2]. However, the long-term prognosis of patients has not been improved. Chronic allograft dysfunction (CAD) is the main cause of graft loss after RT [3,4]. The prominent histopathologic features are interstitial fibrosis and tubular atrophy (IF/TA), with excessive deposition of fibrillar extracellular matrix (ECM) [5,6]. Progression of IF/TA is associated with renal function deterioration. Moreover, moderate-to-severe IF/TA (Grade ≥ 2) occurs in between 17% and 66% of patients after 5 years of renal transplantation [7]. Up to now, renal biopsy is the only reference standard to evaluate IF/TA grades. However, it is limited due to several disadvantages: (1) it is an invasive procedure; (2) sample tissue accounts for <1% of renal tissue, which means the tissue of renal biopsy may not be sufficiently representative in some cases and it is difficult to monitor the development of disease dynamically in real-time; (3) possible complications in up to 2.7%–5.2% of cases [8,9], including gross hematuria (2.2%–3.5%), hematoma (1.1%–7.1%), and urinary fistula (2.7%). Therefore, evaluating IF/TA grades accurately and noninvasively is essential for CAD patients and important for clinical decision-making.

Conventional ultrasound is well-suited to evaluating renal allograft morphologic changes by measuring renal size and cortical echogenicity, but it is limited in detecting diffuse diseases including renal fibrosis. Resistive index (RI), an important parameter measured by color Doppler flow imaging (CDFI), reflects the hemodynamic status of renal allograft. The relationship between RI and renal histopathology is still controversial [10,11,12]. Some studies have reported that RI could act as diagnostic standard of acute rejection (AR) after RT [10,13]. However, Rother et al. [11] found that RI detected neither chronic allograft injury such as IF/TA nor acute rejection and acute tubular necrosis.

Shear wave elastography (SWE) developed rapidly and was used to evaluate tissue stiffness quantitatively. In previous studies, our team demonstrated the reliability of SWE in assessment of liver fibrosis [14] and lower leg muscle stiffness [15]. Point-shear wave elastography (p-SWE), one type of SWE method, generates shear wave on the basis of the acoustic radiation push-pulses of short-duration. The shear wave velocity of the tissue quantitatively acquired by p-SWE in real-time is based on the B-mode imaging. The tissue stiffness can be expressed as Young’s modulus. A faster shear wave velocity and higher Young’s modulus are the mirror of stiffer tissue. At present, p-SWE is recommended for noninvasively evaluating the stages of liver fibrosis by EFSUMB, WFSUMB, and other academic organizations due to its high accuracy and good reproductivity [16,17]. Benefiting from the superficial location, the renal transplant is suitable for measurement by p-SWE [18]. Recent studies have shown the feasible application of p-SWE in evaluating renal fibrosis in renal transplant, but the results are controversial [19,20,21]. Yang, La et al. [22] assessed the different complications after renal transplantation on a sample of 31 patients and reported that the renal stiffness could evaluate moderate-to-severe renal fibrosis (AUC = 0.92, cutoff = 38.71 kPa). However, Juhan Lee [19] considered that renal stiffness measured by p-SWE had no correlation with IF/TA stages, but it was correlated with the time after transplantation. In 2016, Cheng Yang [10] established a novel model involving p-SWE, eGFR, and other markers to discriminate AR with non-AR after renal transplantation in 115 patients. To our knowledge, there was no study combining p-SWE with other parameters for the assessment of IF/TA stages in CAD patients.

Serum biomarkers, such as estimated glomerular filtration (eGFR), have been documented to evaluate the function of transplanted renal. Whether eGFR correlates with renal fibrosis is still not clear. Gungor, Guzel et al. found that eGFR was correlated with fibrosis in patients with diabetic nephropathy, but not in patients with renal transplant [23]. Additionally, Cheng Yang et al. [10] reported that eGFR (AUC = 0.803) could discriminate patients from AR to those without. They also suggested combining eGFR with other parameters had higher diagnosis accuracy. Therefore, it is necessary to search a noninvasive and reliable novel model for assessment of IF/TA stages.

First, this prospective study aims to evaluate the feasibility of p-SWE in assessing different IF/TA stages and further establish a novel and noninvasive diagnostic method for CAD patients. Second, the study aims to explore the correlations between renal stiffness and clinical and pathologic findings. The results of this study are expected to evaluate IF/TA noninvasively, as well as lay the foundations for treatment.

## 2. Materials and Methods

### 2.1. Study Population

**Figure 1:**
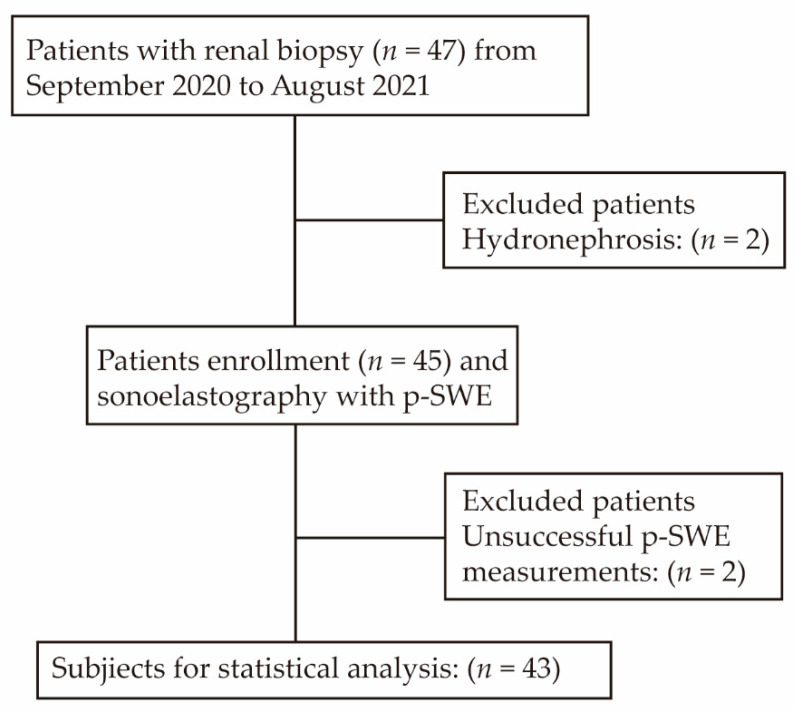
Flow chart of all patients.

Between September 2020 and August 2021, 47 patients with clinical renal dysfunction who planned or decided to undergo ultrasound-guided renal biopsy were consecutively enrolled in this study. Additionally, all patients consented to p-SWE examinations. The results of conventional ultrasound, Doppler ultrasound, p-SWE, serum biomarkers, and biopsy for each patient were collected. The time intervals between renal biopsy and p-SWE were less than 7 days. Patients with CAD were defined as having worsened renal function for more than 3 months without recovery (eGFR < 50 mL/min/1.73 m^2^, increased serum creatinine). The inclusion criteria were as follows: (1) patients defined as chronic renal allograft dysfunction who planned or decided to undergo renal biopsy; (2) over 18 years of age; (3) more than 1 year since renal transplantation. The exclusion criteria were as follows: (1) companion with other diseases, including hydronephrosis and renal artery stenosis; (2) unsuccessful p-SWE measurements. Thus, 43 patients were finally enrolled for statistical analysis (Figure 1). All the patients provided informed consent to participate, and The Third Medical Center of Chinese PLA General Hospital review board approved this prospective study. No data were included that were sourced from executed prisoner’s donations, and the procedure in this study was designed in accordance with the Declaration of Helsinki. The demographic and clinical data of the patients (age, sex, body mass index, and underlying diseases) were recorded.

### 2.2. Serum Biomarkers

Blood counts and renal function tests were performed 1 week before renal biopsy. The urea level, serum creatinine level, proteinuria, eGFR (mL/min/1.73 m^2^), white blood cell (WBC) count, red blood cell (RBC) count, and platelet (PLT) count were recorded. Using the Modification of Diet in Renal Disease Study equation, the estimated glomerular filtration rate (eGFR) was calculated: eGFR (female) = 186 × SCr − 1.154 × age − 0.203 × 0.742, and eGFR (male) = 186 × SCr − 1.154 × age − 0.203, where SCr = serum creatinine.

### 2.3. Ultrasound and p-SWE Examinations

**Figure 2:**
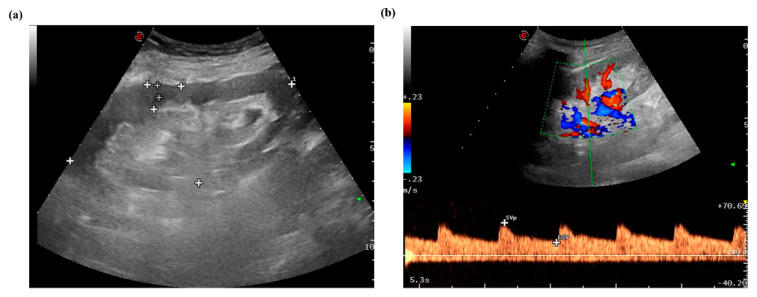
(**a**) B-mode image, measurement of renal size including renal length, width, and renal parenchyma thickness. The plus signs mean activating the measurement key after freezing the ultrasound imagine and the green color means focal position.(**b**) CDFI image, measurement of resistive index (RI).

The patients were evaluated with B-mode imaging, Doppler ultrasound and p-SWE measurements 1 week before biopsy. The measurement procedure was performed by using the software Esaote(version MyLab 8Exp, Genova, Italy), and a convex broadband probe (C1–8). All measurements were obtained by an ultrasound sonographer (Y.G) who was blinded to biopsy results. The sonographer has more than 10 years of ultrasound experience and more than 200 shear wave elastography examinations. The size of the transplanted kidney (length and width) and skin-allograft distance were measured by B-mode imaging (Figure 2a). CDFI was used to evaluate blood perfusion of the transplanted renal, such as resistance index (RI) (Figure 2b). 

**Figure 3:**
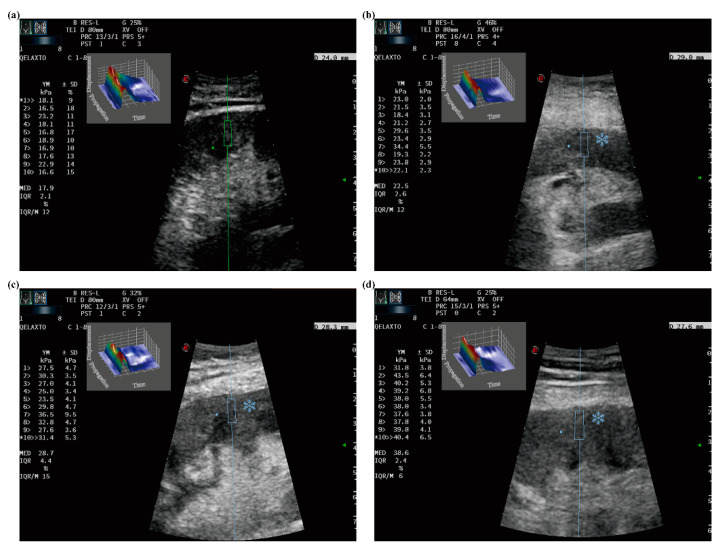
Measurements of p-SWE in renal transplants with different IF/TA stages: (**a**) IF/TA Grade 0, median = 17.9 kPa; (**b**) IF/TA Grade 1, median = 22.5 kPa; (**c**) IF/TA Grade 2, median = 28.7 kPa and (**d**) IF/TA Grade 3, median = 38.6 kPa. The line is used to drive operator to select suitable measurement areas. The square showed 3D eWave which is an exclusive tool that provides immediate feedback about the quality of the shear wave produced in the tissue, and is the three-dimensional representation of the shear waves generated by p-SWE. The asterisk showed the cooling indication (2 s in average) which is directly on the measurement site for “eye focus” feedback.

The p-SWE technique is characterized by the generation of the shear wave propagation to obtain tissue stiffness. Before the measurements, patients were suggested to practice breathing. During the p-SWE examinations, patients were required to hold their breath for approximately 3–4 s in a natural breathing state and lay in the supine position. To avoid the influence of probe pressure, the ultrasound transducer was placed vertically on the surface of the transplanted kidney and compressed slightly. A 5 mm × 10 mm region of interest (ROI) was placed in a parenchyma-area free of vessels in the p-SWE image. Ten repetitive and consecutive measurements (lasting 4–6 min) were performed in the same site. The median (M), interquartile range (IQR), and IQR/M of the elasticity values were automatically calculated and displayed. The median of 10 elasticity measurements in kilopascals was used for statistical analysis. Measurements were considered to fail when IQR/M was >30% (Figure 3).

### 2.4. Renal Biopsy and Histologic Assessment

US-guided percutaneous renal biopsy was performed in the renal parenchyma with a 16- or 18-gauge automated edge-cutting biopsy needle. Specimens obtained from renal biopsy were stained with hematoxylin-eosin and Masson trichrome. Only biopsies at least 10 mm in length and with 2 sclerosing glomeruli were considered. All biopsies were graded using Banff scores by scoring the immunological parameters, including the mannose binding lectin pathway (C4 d), peritubular capillaritis (ptc), glomerulitis (g), interstitial inflammation (i), tubulitis (t), total interstitial inflammation (ti), intimal arteritis (v), arteriolar hyaline thickening (ah), hyaline arteriolar thickening (aah), interstitial fibrosis (ci), tubular atrophy (ct), fibrous intimal thickening (cv), mesangial matrix increase (mm), and allograft glomerulopathy (cg). The IF/TA grade was calculated based on the ci and ct scores. The histological diagnosis, including the IF/TA grade, was evaluated by an experienced pathologist (with more than 10 years of experience), who was blinded to the p-SWE measurements. 

### 2.5. Statistical Analysis

Quantitative variables were expressed as medians and IQRs, and qualitative variables were expressed as counts and percentages. Correlations between p-SWE and baseline parameters were analyzed using the Pearson correlation, and correlations between p-SWE and pathological findings were analyzed using the Spearman correlation. The correlation coefficient was classified as follows [24]: poor correlation <0.4; moderate correlation 0.4–0.75; strong correlation >0.75. Univariate and multivariate analyses were used to identify correlated factors for different IF/TA stages. The diagnostic values of IF/TA stages were evaluated by calculating AUCs. Differences between the AUCs were compared using a DeLong test. Cutoff values, sensitivity, specificity, positive and negative predictive values were computed. The intraclass correlation coefficient (ICC) was calculated to assess agreement for p-SWE measurements. The data were analyzed by using SPSS software for Windows (version 22.0; SPSS, Chicago, I11) and MedCalc software (version 15.2; MedCalc, Mariakerke, Belgium). All statistical tests were two-sided and *p* < 0.05 indicated a significant difference or correlation.

## 3. Results

### 3.1. Baseline Characteristics

From September 2020 to August 2021, 47 eligible patients were enrolled in this study. Among them, four patients were excluded because of hydronephrosis, unsuccessful p-SWE measurements and no consent. Thus, 43 patients were finally enrolled for statistical analysis. We also collected data about donor type including deceased donor (*n* = 36, percent 83.7%) and living donor (*n* = 7, percent 16.3%). The baseline characteristics of the enrolled patients are shown in Table 1. The study consisted of 38 males and 5 females, with a median age of 43 years. Hypertension (*n* = 21, percent 48.8%) was the most common underlying disease. The median time since transplantation was 48 months (range, 28 to 64 months), and the median renal stiffness was 23.1 kPa (range, 20.4 to 25.1 kPa), as measured by p-SWE. The intraclass correlation coefficient (ICC) of ten p-SWE measurements was 0.64 (95%CI: 0.51–0.77). The IF/TA grades were based on Banff 2007 and graded from Grade 0 to Grade 3 [25] (Table 2). The histopathological findings on IF/TA were as follows: 12 patients were classified as IF/TA Grade 0 (27.9%); 17 patients were classified as IF/TA Grade 1 (39.5%); 10 patients were classified as IF/TA Grade 2 (23.3%); and 4 patients were classified as IF/TA Grade 3 (9.3%).

### 3.2. Comparison Renal Cortical Stiffness, eGFR, Proteinuria and Resistive Index in Patients between IF/TA Grade 0–1 and Grade 2–3

**Figure 4:**
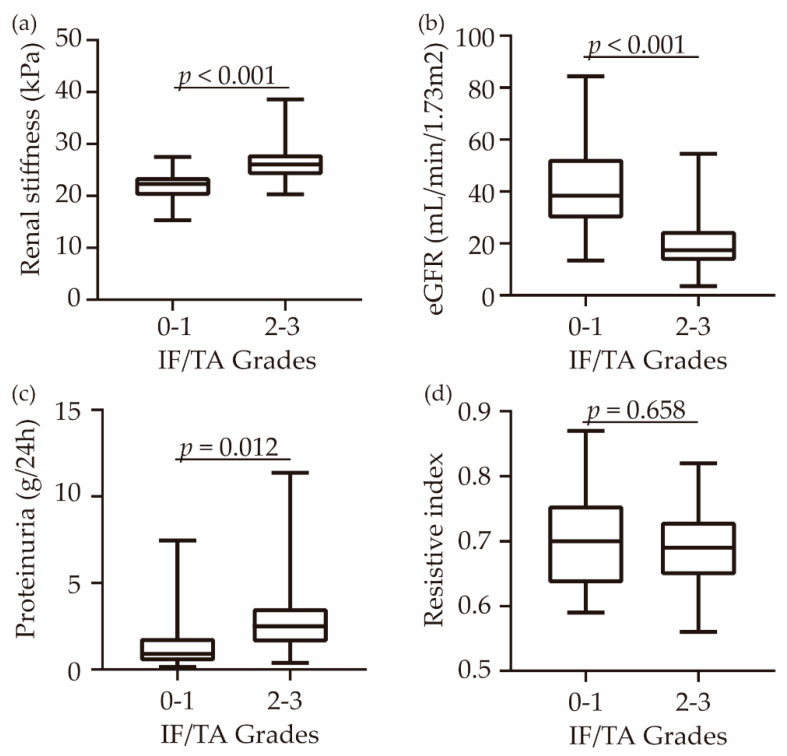
Comparison of renal function markers and p-SWE measurements between IF/TA Grade 0–1 and IF/TA Grade 2–3. The renal cortical stiffness (**a**), eGFR (**b**), proteinuria (**c**), and resistive index (**d**) were compared. The error bars are the minimum and maximum values. The lines through the middle of the boxes represent the median. The central box represents the interquartile range.

All the patients were divided into an IF/TA Grade 0–1 group and Grade 2–3 group. Renal cortical stiffness and proteinuria of IF/TA Grade 2–3 were significantly higher than Grade 0–1 (*p* < 0.001, *p* = 0.012, respectively) (Figure 4a,c). The eGFR of IF/TA Grade 2–3 was lower than IF/TA 0–1 (Figure 4b). In addition, the RI of the two groups showed no difference (*p* = 0.658) (Figure 4d). Spearman’s correlation coefficient for renal stiffness measurements and IF/TA grades was 0.713 (*p* < 0.001).

### 3.3. Correlations between Renal Cortical Stiffness and Baseline Parameters

Renal cortical stiffness showed a significantly moderate positive correlation with proteinuria (r = 0.526, *p* < 0.001), and was the best correlation among the serum biomarkers. Additionally, renal cortical stiffness measured by p-SWE was poor correlated with urea (r = 0.394, *p* = 0.009), serum creatinine (r = 0.372, *p* = 0.014), and inversely related to the eGFR (r = −0.375, *p* = 0.013). However, there was no correlation between renal cortical stiffness and other characteristics including age, sex, BMI, time since transplantation, RI and skin-allograft distance (Table 3).

### 3.4. Correlations between Renal Cortical Stiffness and Histopathological Findings

The pathological examinations reveal other significant findings such as chronic rejection, drug-induced renal injury and BK virus nephropathy. In addition, the median number of glomeruli per biopsy was 13, range 11–21. For the 43 enrolled patients, the histopathological findings were based on the semiquantitative Banff classification. Renal cortical stiffness showed positive correlations with i (r = 0.494, *p* = 0.001), t (r = 0.462, *p* = 0.002), ti (r = 0.535, *p* < 0.001), ci (r = 0.563, *p* < 0.001) and ct (r = 0.649, *p* < 0.001). With the increasing of renal stiffness, the degree of chronic lesions after RT including i, t, ti, ci and ct was deepened. However, it did not correlate with other chronic lesions including C4d, ptc, g, v, ah, aah, cv, mm, and cg (Table 4).

### 3.5. Analysis of Factors Associated with Moderate-to-Severe IF/TA

Univariate logistic regression showed that age (OR = 0.93, *p* = 0.036), eGFR (OR = 0.92, *p* = 0.007), proteinuria (OR = 1.46, *p* = 0.040), and renal stiffness (OR = 1.98, *p* = 0.003) were associated with moderate-to-severe IF/TA (grade ≥ 2). Renal stiffness (OR = 1.78, *p* = 0.012) and eGFR (OR = 0.93, *p* = 0.049) were independent predictors for moderate-to-severe IF/TA by multivariate logistic regression analysis (Table 5). In line with the results of multivariate regression analysis, a new model that combined renal stiffness and eGFR was established. The constant from the multivariate analysis by binominal logistic regression was −11.061, and the formula was as follows:

$$y=-11.061+0.542\times \;\mathrm{Renal}\;\mathrm{stiffness}\left(\mathrm{kPa}\right)-0.084\times \;\mathrm{eGFR}\left(\frac{\frac{\mathrm{mL}}{\min}}{1.73}2\right)$$

### 3.6. Assessment Diagnosis of Histopathological Banff Scores 

**Figure 5:**
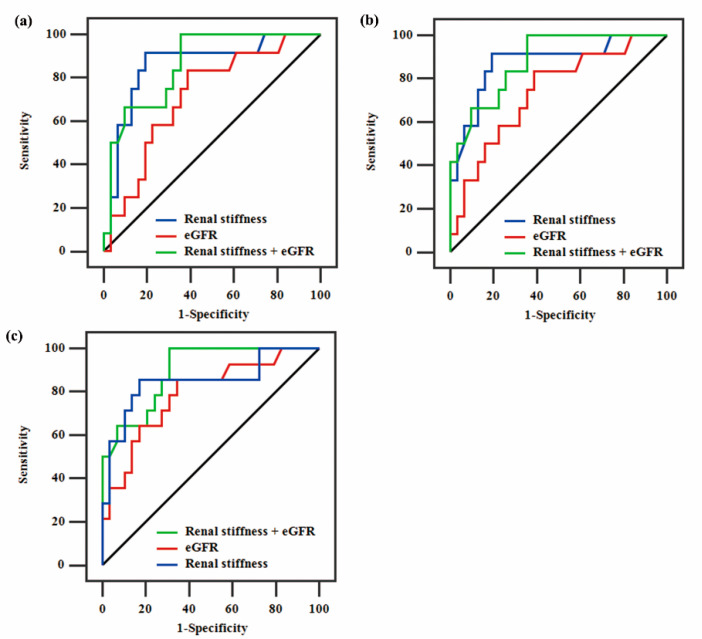
Graph showing receiver operating characteristic curves of p-SWE, eGFR, and p-SWE plus eGFR in the diagnosis of: (**a**) moderate-to-severe ci, (**b**) moderate-to-severe ct, and (**c**) moderate-to-severe IF/TA.

Based on the Banff scores of 0–3 points, patients with 0 and 1 point were combined, and patients with 2 and 3 points were also combined to evaluate diagnostic accuracy. The AUCs of p-SWE, eGFR, and p-SWE plus eGFR were 0.86, 072, and 0.86 for moderate-to-severe ci (grade ≥ 2) (Figure 5a), and 0.87, 0.74, and 0.88 for moderate-to-severe ct (grade ≥ 2) (Figure 5b), respectively. In addition, the diagnostic accuracy of p-SWE, eGFR, and p-SWE plus eGFR in assessing moderate-to-severe IF/TA (grade ≥ 2) was determined (Figure 5c). The comparison of AUCs revealed that p-SWE was similar to eGFR in evaluating moderate-to-severe IF/TA (AUC = 0.85 and 0.79, *p* = 0.61). Using p-SWE plus eGFR did increase the diagnosis of identifying moderate-to-severe IF/TA (AUC = 0.89). Cutoff values of the p-SWE, eGFR, and p-SWE plus eGFR with sensitivity, specificity, PPV, and NPV are summarized in Table 6. The optimal cutoff values were determined by analysis of the AUCs based on the Youden index.

## 4. Discussion

IF/TA is a dynamic and progressive process, and moderate-to-severe IF/TA is a significant risk factor leading to renal graft loss [5]. The early, noninvasive assessment of IF/TA stages is important for clinical decision-making. B-mode images, CDFI, and serum biomarkers are still routinely used for monitoring renal allografts. p-SWE, as a noninvasive technology, has been used to evaluate renal allograft fibrosis. The current study showed that the combination of p-SWE and eGFR has good diagnostic accuracy in assessing moderate-to-severe ci, ct, and IF/TA. The corresponding AUCs were 0.86, 0.88, and 0.89. Additionally, the combination of p-SWE and eGFR is significantly superior to eGFR alone in assessing moderate-to-severe ci and ct, respectively. 

The RI is mainly used to diagnose rejection after transplantation. However, in our study, the mean RI of all patients was 0.69 and showed no significant difference between IF/TA Grade 0–1 and Grade 2–3 (*p* = 0.658). Likewise, Bom iun kim [24] found that RI was not different between subclinical rejection (SCR) and without SCR groups. These results suggested that RI may not be an effective parameter to evaluate IF/TA stages. eGFR is another parameter usually applied in the evaluation of renal allograft status. In that study [24], eGFR was a significantly independent predictor of subclinical rejection in univariate and multivariate analysis. We found that the eGFR of IF/TA Grade 2–3 was lower than Grade 0–1 and that eGFR was also significantly correlated with moderate-to-severe IF/TA both in univariate and multivariate analysis (*p* < 0.05). Moreover, Cheng Yang [10] et al. reported that combining eGFR with p-SWE and other parameters had higher diagnosis accuracy in discriminating patients from AR to non-AR. Therefore, these findings prompted us to establish a better index in CAD patients.

p-SWE technology is a well-established noninvasive method for the assessment of liver fibrosis. In recent years, it has been applied to the evaluation of renal transplant, with the following advantages: (1) good reproducible measurement and high intra- and inter-observer agreement, with previous studies showing that the ICC value of p-SWE is from 0.84 to 0.90 [20,26]; (2) application of different diseases after renal transplantation, with the first clinical experience using p-SWE suggesting that it is a complementary method in the diagnosis of renal allograft rejection [27] and other studies also finding that p-SWE technology can be used to determine acute rejection after renal transplantation [10,13] and to evaluate renal fibrosis [26]; and (3) real-time measurement of tissue stiffness. Thus, p-SWE technology was used to measure the renal stiffness in this study. Overall, 45 patients were successfully measured by p-SWE, while it failed in two patients, demonstrating a success rate of 95.6%. 

In addition, this study found that there was no correlation between p-SWE and demographic parameters including age, sex, and BMI. Nicolas Grenier observed that renal stiffness measured by supersonic shear imaging (SSI) had no correlation with baseline characteristics (*p* > 0.05) [28], which is consistent with our results. However, Järv et al. [29] reported that renal stiffness had negative correlations with age (r = −0.2, *p* = 0.03) and BMI (r = −0.3, *p* = 0.01). The reason why their result is different from ours may be that Järv et al. included more patients who were more than 5 years post renal transplantation, a group which only accounted for 30% of the patients in our study. We also noted that the renal stiffness showed a negative correlation with eGFR (r = −0.375, *p* = 0.013). This means that renal stiffness might reflect the functional state of renal allografts. The results were also confirmed in other techniques, such as 2-dimension shear wave elastography (2D-SWE) and transient elastography (TE) [12,30,31]. Specifically, Nitin P. Ghonge observed that SWE had a moderate positive correlation with RI (r = 0.56, *p* < 0.001) [30], whereas we observed that renal stiffness had no correlation with RI (r = 0.172, *p*= 0.277). The reason for the difference might be the inconsistent enrolled population. In our study, we only counted patients with renal biopsies, while Nitin P. Ghonge’s study enrolled all patients with stable allograft function who did not undergo biopsy. In addition, a pilot study showed that renal stiffness using SSI was not correlated with any single semiquantitative Banff score [28]. However, our findings suggested that renal stiffness had positive correlation with some pathological scores, especially with interstitial fibrosis (r = 0.591, *p* <0.001) and tubular atrophy (r = 0.653, *p* < 0.001).

To our knowledge, this is the first study to combine p-SWE with eGFR to evaluate IF/TA grades in patients with CAD. However, the limitations of this study need to be noted. First, the number of patients enrolled in our study was small. Only 43 patients (including five female patients, accounting for 11.6%) who underwent renal biopsies were included, and patients with IF/TA Grade 3 were relatively few (five patients with IF/TA Grade 3, a percentage of 9.3%). The unbalanced gender distribution might be coincidental, due to the existence of a random sampling error. This present study is prospective, and more studies are needed to reflect a more balanced patient distribution in IF/TA grades and sex, so as to increase the population and improve diagnosis accuracy. Second, our study is an attempt to use p-SWE combined with eGFR for evaluating IF/TA grades. In fact, biopsies provide other important and comprehensive pathological analysis, such as inflammation and glomerular changes. We consider that IF/TA is the main aspect that affects stiffness. Of course, other aspects may also affect stiffness. Due to the limitation of sample size, this method cannot replace biopsy. Further studies should expand the sample size needed to assess inflammation and other pathological findings. Finally, the kidneys make up less than 1% of the body weight but renal blood flow accounts for 20% of cardiac output. The renal microcirculation hemodynamics may also influence renal function and renal stiffness [32]. CEUS, a novel ultrasound technology, has been identified to evaluate microcirculation blood flow quantitatively. Further studies could consider combining p-SWE with CEUS to noninvasively explore the evaluation of IF/TA stages by multiple ultrasound technologies.

## 5. Conclusions

In conclusion, all of these results suggested p-SWE might become a noninvasive tool for evaluating IF/TA stages in CAD patients. In addition, based on these results, we propose that p-SWE combined with eGFR may increase diagnostic accuracy in assessing moderate-to-sever interstitial fibrosis or tubular atrophy. Therefore, p-SWE combined with eGFR appears to be a feasible and promising method for assessing IF/TA stages.

## Figures and Tables

**Table 1:** Patients’ characteristics.

Characteristics	Value
Donor	
Donor type ^1^	
Deceased donor	36 (83.7%)
Living donor	7 (16.3%)
Donor age, years ^2^	52 (48–59)
Recipient	
Age, years	43 (38–56)
Male/Female	38/5 (88.4%/11.6%)
BMI	24.2 (22.6–26.9)
Underlying disease	
No	9 (20.9%)
Hypertension	21 (48.8%)
Diabetes	5 (11.6%)
Hypertension and diabetes	8 (18.6%)
Time since transplantation (months)	48 (28–64)
Immunosuppressor	
Tacrolimus	38 (88.4%)
Cyclosporine	5 (11.6%)
Steroids	37 (86.1%)
Biomarkers	
Urea (mmol/L)	14.61 (10.89–20.18)
Serum creatinine (mg/dL)	2.35 (1.71–3.41)
Proteinuria (g/24 h)	1.30 (0.61–2.51)
eGFR (mL/min/1.73 m^2^)	31.2(19.6–47.8)
WBC	6.62 (4.95–8.54)
RBC	3.71 (3.24–4.41)
PLT	201.00 (158.75–231.00)
Ultrasound examinations	
Renal length (cm)	11.39 (10.72–12.10)
Renal width (cm)	5.30 (4.76–5.80)
RI	0.69 (0.64–0.73)
Skin-allograft distance (cm)	2.12 (1.57–2.58)
Grade of IF/TA	
0	12 (27.9%)
1	17 (39.5%)
2	10 (23.3%)
3	4 (9.3%)
Renal stiffness (kPa)	23.1 (20.4–25.1)

^1^ Categorical variables are expressed as frequencies (percentages). ^2^ Continuous variables are expressed as median (IQR).

**Table 2:** Banff classification of IF/TA grades.

Grades	Definition
Grade 0	No interstitial fibrosis
Grade 1	Mild interstitial fibrosis, <25%of the cortical area
Grade 2	Moderate interstitial fibrosis, 26–50% of the cortical area
Grade 3	Severe interstitial fibrosis, >50% of cortical area

**Table 3:** Correlations between renal stiffness and baseline parameters.

Variables	r	*p* Value
Age	−0.170	0.276
Sex	−0.047	0.766
BMI	−0.098	0.531
Time since transplantation	−0.104	0.505
Urea (mmol/L)	0.394	0.009
Serum creatinine (mg/dL)	0.372	0.014
Proteinuria (g/24 h)	0.526	<0.001
eGFR (mL/min/1.73 m^2^)	−0.375	0.013
RI	0.198	0.203
Skin–allograft distance (cm)	−0.005	0.973

**Table 4:** Correlations between renal cortical stiffness and histological findings.

	r	*p* Value
Banff score		
C4d	0.080	0.612
ptc	0.055	0.726
g	−0.151	0.333
i	0.494	0.001
t	0.462	0.002
ti	0.535	<0.001
v	0.168	0.282
ah	−0.068	0.663
aah	0.018	0.911
ci	0.563	<0.001
ct	0.649	<0.001
cv	0.207	0.184
mm	0.032	0.839
cg	−0.033	0.834
IF/TA	0.662	<0.001

Abbreviations: C4d, the mannose binding lectin pathway; ptc, peritubular capillartis; g, glomerulitis; i, interstitial inflammation; t, tubulitis; ti, total interstitial inflammation; v, intimal arteritis; ah, arteriolar hyaline thickening; aah, hyaline arteriolar thickening; ci, interstitial fibrosis; ct, tubular atrophy; cv, fibrous intimal thickening; mm, mesangial matrix increase; cg, allograft glomerulopathy. IF/TA interstitial fibrosis and tubular atrophy.

**Table 5:** Results of logistic regression analysis on moderate-to-severe IF/TA (grade ≥ 2).

Parameters	Univariate		Multivariate	
	OR (95%CI)	*p* Value	OR (95%CI)	*p* Value
Sex	0.69 (0.10–4.70)	0.707		
BMI	0.85 (0.69–1.05)	0.149		
eGFR	0.92 (0.87–0.98)	0.007	0.93 (0.86–1.00)	0.043
RI	0.05(0.00–544.13)	0.522		
Proteinuria (g/24 h)	1.46 (1.01–2.10)	0.040	1.21 (0.74–1.99)	0.455
Renal stiffness	1.69 (1.18–2.42)	0.004	1.70 (1.14–2.53)	0.010

**Table 6:** Diagnostic performance of p-SWE, eGFR, and p-SWE combined with eGFR in moderate-to-severe ci, ct, and IF/TA (Grade ≥ 2).

	AUC (95%CI)	Cut-Off	Sensitivity (%)	Specificity (%)	PPV (%)	NPV (%)
Ci						
p-SWE	0.86 (0.72–0.95)	23.7	91.7	80.6	64.7	96.2
eGFR	0.72 (0.56–0.84)	31.2	83.3	61.3	45.5	90.5
p-SWE plus eGFR	0.86 (0.72–0.95)	−1.63	100.0	64.5	52.2	100.0
Ct						
p-SWE	0.87 (0.74–0.96)	23.7	91.7	80.7	64.7	96.2
eGFR	0.74 (0.58–0.86)	31.2	83.3	63.3	45.5	90.5
p-SWE plus eGFR	0.88 (0.75–0.96)	−1.63	100.0	64.5	52.2	100.0
IF/TA						
p-SWE	0.85 (0.71–0.94)	23.7	85.7	82.7	70.6	92.3
eGFR	0.79 (0.64–0.90)	31.2	85.7	65.5	54.5	90.5
p-SWE plus eGFR	0.89 (0.76–0.97)	−1.63	100.0	69.0	60.9	100.0
